# C57BL/6N mice show a sub-strain specific resistance to the psychotomimetic effects of ketamine

**DOI:** 10.3389/fnbeh.2022.1057319

**Published:** 2022-11-24

**Authors:** Zofia Harda, Klaudia Misiołek, Marta Klimczak, Magdalena Chrószcz, Jan Rodriguez Parkitna

**Affiliations:** Department of Molecular Neuropharmacology, Maj Institute of Pharmacology, Polish Academy of Sciences, Kraków, Poland

**Keywords:** ketamine, schizophrenia, social behavior, locomotion, C57BL/6 mice

## Abstract

Repeated administration of subanesthetic doses of ketamine is a model of psychosis-like state in rodents. In mice, this treatment produces a range of behavioral deficits, including impairment in social interactions and locomotion. To date, these phenotypes were described primarily in the Swiss and C3H/HeHsd mouse strains. A few studies investigated ketamine-induced behaviors in the C57BL/6J strain, but to our knowledge the C57BL/6N strain was not investigated thus far. This is surprising, as both C57BL/6 sub-strains are widely used in behavioral and neuropsychopharmacological research, and are *de facto* standards for characterization of drug effects. The goal of this study was to determine if C57BL/6N mice are vulnerable to develop social deficits after 5 days withdrawal from sub-chronic ketamine treatment (5 days, 30 mg/kg, i.p.), an experimental schedule shown before to cause deficits in social interactions in C57BL/6J mice. Our results show that sub-chronic administration of ketamine that was reported to cause psychotic-like behavior in C57BL/6J mice does not induce appreciable behavioral alterations in C57BL/6N mice. Thus, we show that the effects of sub-chronic ketamine treatment in mice are sub-strain specific.

## Introduction

Repeated administration of subanesthetic doses of ketamine is a widely used model of psychosis-like state in rodents (Neill et al., 2010; Frohlich and Van Horn, 2014). Psychotomimetic effects of ketamine are attributed to the antagonism of N-methyl-D-aspartate (NMDA) receptors (Nowacka and Borczyk, 2019). In humans, acute administration of ketamine induces psychosis-like symptoms, including hallucinations and impaired cognitive abilities (Krystal et al., 1994). In mice, sub-chronic (5–24 days) administration of subanesthetic (5–100 mg/kg) doses of ketamine produces deficits in social interactions (Vasconcelos et al., 2015; da Silva Araújo et al., 2017; Onaolapo et al., 2017; Ogundele and Lee, 2018; Sultana and Lee, 2020), pre-pulse inhibition (Monte et al., 2013; Vasconcelos et al., 2015; da Silva Araújo et al., 2017), auditory processing (Maxwell et al., 2006; Amann et al., 2009), and cognition (Featherstone et al., 2012). This treatment also induces despair-like behavior (Chatterjee et al., 2011, 2012; Choudhury et al., 2016) and locomotor hyperactivity (Monte et al., 2013; Vasconcelos et al., 2015; Choudhury et al., 2016; da Silva Araújo et al., 2017; Onaolapo et al., 2017) (though hypoactivity was also reported; Sultana and Lee, 2020). Ketamine effects have been studied at different time points after cessation of drug treatment (up to 6 months). Some of the studies reported altered behaviors over extended periods of time (Chatterjee et al., 2011; Featherstone et al., 2012). To date, these effects were described mainly in the Swiss mouse strain (Chatterjee et al., 2012; Monte et al., 2013; Vasconcelos et al., 2015; Choudhury et al., 2016; da Silva Araújo et al., 2017; Onaolapo et al., 2017; Ben-Azu et al., 2018) and C3H/HeHsd mice (Maxwell et al., 2006; Featherstone et al., 2012). Relatively few studies employed C57BL/6 mice: the C57BL/6J strain (Amann et al., 2009; Sultana and Lee, 2020), C57BL/6Hsd (Maxwell et al., 2006), and in some cases the sub-strain was not specified (Choudhury et al., 2016; Ogundele and Lee, 2018).

The goal of this study was to determine if C57BL/6N mice are vulnerable to develop social deficits after 5 days withdrawal from sub-chronic ketamine treatment. To induce behavioral changes, we used a ketamine administration scheme shown before to cause deficits in social interactions in C57BL/6J mice (Sultana and Lee, 2020). Furthermore, we wanted to determine if a selective kappa opioid receptor antagonist, norbinaltorphimine, could affect behaviors induced by sub-chronic ketamine administration. This part of the study was prompted by the observation that nonselective kappa opioid receptor ligands (e.g., naltrexone and buprenorphine) have immediate antipsychotic effects in schizophrenic patients, and by the recent hypothesis that kappa opioid antagonists could be effective in the treatment of schizophrenia (Clark and Abi-Dargham, 2019).

## Materials and methods

### Animals

Male C57BL/6N mice from Charles River Laboratories (Germany) aged 12 weeks at the start of the procedures were used as subject animals (later referred to as “actors”). Male C57BL/6J mice aged 7 weeks from the colony at the Maj Institute of Pharmacology Polish Academy of Sciences were used as stimulus animals (later referred to as “partners”). Actors arrived at the Maj Institute of Pharmacology 2 weeks prior to the experiment. Mice were handled by the experimenter for 4 days before the experiment. Both actors and partners were group-housed (three to four animals per cage) in a normal dark-light cycle (dark 6 p.m.–6 a.m.), in cages containing gnawing blocks and nesting material, with food and water *ad libitum*. Drug injections and behavioral testing were performed during the light phase, as was done in the previous study that reported social deficits in C57BL/6J mice after withdrawal from sub-chronic ketamine injections (Sultana and Lee, 2020). The tests started 1 h into the light phase or later, and were completed no later than 1 h before the onset of the dark phase. Prior studies that investigated the impact of the testing phase on the results of social interaction tests show that behavioral scores obtained in the dark and light phases are similar (Yang et al., 2008). All procedures were performed in accordance with the European Union (directive no. 2010/63/UE) and Polish regulations concerning animal research. Experimental plan was pre-approved by the 2nd Local Ethics Committee in Krakow, Poland (permit number 306/2020). No animals were excluded from the study due to health issues.

### Drugs and treatment schedule

**FIGURE 1:**
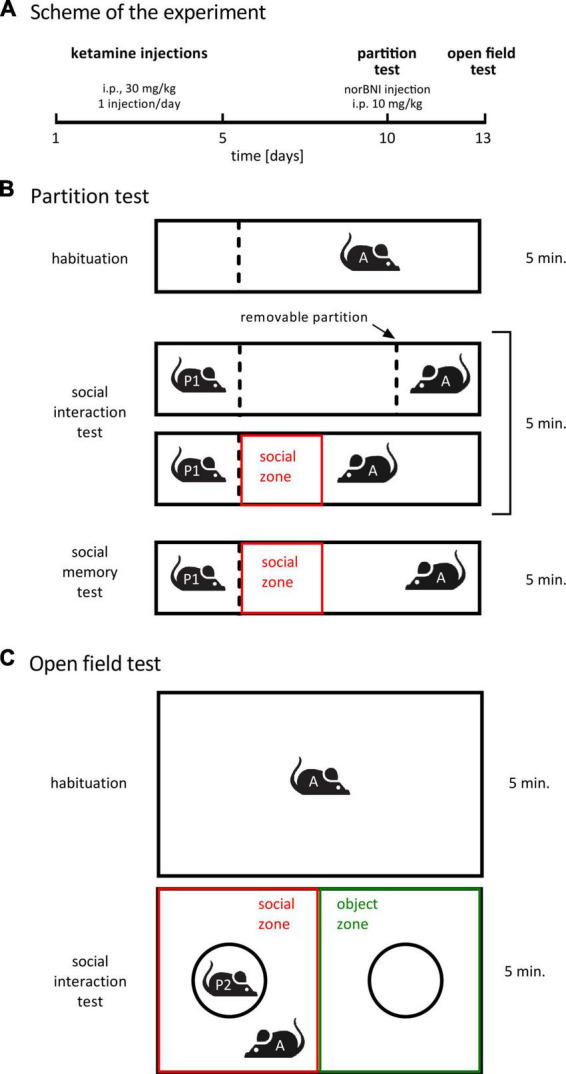
Treatment schedule and behavioral paradigms. **(A)** A summary of the treatment schedule. norBNI indicates norbinaltorphimine. **(B)** A scheme of the partition test. The dotted lines represent a transparent perforated Plexiglas wall. The red box shows the area that was designated as social interaction zone during video analysis. “A” stands for “actor” (subject animal), “P” stands for “partner”. **(C)** A summary of the open field test. The circles represent wire cups. The red and green rectangles represent, respectively, the social zone and the object zone during video analysis.

Experimental schedule was taken from (Ogundele and Lee, 2018; Sultana and Lee, 2020). Mice were randomly allocated to the three treatment groups listed in Table 1 (*n* = 8 for each group, i.e., 24 animals in total). Ketamine (ketamine hydrochloride, Biowet, Poland) was administered once a day for 5 days (i.p., 30 mg/kg, 5 μl/g). Five days after the last injection social interactions with a novel conspecific were assessed in the partition test (Figure 1A). Four hours before the test, animals received norbinaltorphimine (norbinaltorphimine dihydrochloride, 0347, Tocris, UK) injection (i.p., dissolved in saline, 10 mg/kg, 5 μl/g). Three days after the partition test, social interaction with an unfamiliar conspecific placed under an enclosure was measured in the open field. Norbinaltorphimine was reported to act for up to 2 weeks (Lalanne et al., 2017), thus a single injection is sufficient to achieve persistent effects during both behavioral tests, performed 3 days apart.

**TABLE 1:** Treatment groups.

Group name	Weight[g] shown as mean (SEM)	Phase 1: Psychotic-like symptoms induction	Phase 2: Psychotic-like symptoms rescue
Sal sal	25.58 (0.35)	Saline	Saline
Ket sal	25.38 (0.21)	Ketamine i.p., 30 mg/kg, 5 μl/g	Saline
Ket norBNI	25.46 (0.48)	Ketamine i.p., 30 mg/kg, 5 μl/g	Norbinaltorphimine, i.p., 10 mg/kg, 5 μl/g

### Partition test: Interaction with a novel conspecific behind the wall

The cage design and test were inspired by Kudryavtseva (2003) and Langford et al. (2010). One day before the test, partner animals were habituated to partner’s compartment for 6 min. The test consisted of three phases: habituation, sociability test (trial 1) and social memory test (trial 2), 5 min each (Figure 1B). The testing cage (length: 50 cm; width: 12 cm; height: 24.5 cm) was divided into different compartments through transparent plastic walls. In the habituation phase, the cage was divided into two compartments by one plastic wall: a smaller compartment (1/4 of the cage) on the left, which was designated to be the partner’s compartment during the second phase, and a larger compartment (3/4 of the cage), in which the actor was released. The wall dividing the compartments was transparent and perforated, so that during the test phase animals could see and smell, but not touch each other. During habituation, the actor was able to move freely in the larger compartment, but was not able to enter partner’s compartment. After habituation, the actor animal was moved to a transport cage for a short time. During this time, a second plastic wall was introduced to the cage, separating the small compartment (1/4 of the cage) on the right, opposite to the partner’s compartment, from the rest of the cage. This second small compartment is referred to as the actor’s compartment. Finally, in the test phase, a partner mouse (unfamiliar adult C57BL/6J male) was placed in the partner’s compartment. Next, the actor was immediately placed in the actor’s compartment and the transparent wall separating the actor from the rest of the cage was lifted, while the transparent wall separating the partner’s compartment from the rest of the cage was kept in its place. Latency to the first approach to the partner’s compartment was measured. After the first trial and 30 min of inter-trial interval, a second 5 min trial with the same partner animal was performed. The aim of the second trial was to measure social memory (Donegan et al., 2020). Reduction of time spent investigating a partner animal on the second vs. first encounter is regarded as an indicator of social recognition. We compared time spent in social zone (the 12.5 cm × 12 cm rectangular zone close to the partner’s compartment) between the first and second trial of the partition test. No difference in the time spent in social zone during the first compared to second trial was observed in any of the groups, i.e., the test did not show any indication of social memory. Thus, the results of the second trial are not shown.

### Open field test: Interaction with a novel confined conspecific

Open field test was performed as previously published (Ogundele and Lee, 2018; Sultana and Lee, 2020). The test constituted of two phases: habituation and social interaction test, 5 min each (Figure 1C). For habituation, the tested mouse was introduced into an empty open field arena open field arena (length: 55 cm; width: 37.5 cm; height: 20.5 cm). Next, two wire cups (9 cm diameter, B-0197, Bionovo, Poland) were placed in the cage: one covering an unfamiliar conspecific (adult C57BL/6J male) and one empty (as a novel object).

### Data analysis

Locomotor activity and time spent in social zone during partition test, and time spent in social and object zones during open field test were measured automatically with EthoVision 15 software (Noldus, The Netherlands). In the partition test, the social zone was defined as a 12 ×12.5 cm rectangular adjacent to the partner’s compartment (Figure 1B). In the open field test social and object zones were defined as half of the cage containing a cup with a partner or an empty cup, respectively (Figure 1C). Zones were defined digitally, no physical barriers between the zones were present in the experimental cages. Time to the first approach to the partner’s zone in partition test and time spent investigating the cups in the open field test were scored manually using the BORIS software package (Friard and Gamba, 2016) by observers blind to the treatment.

**FIGURE 2:**
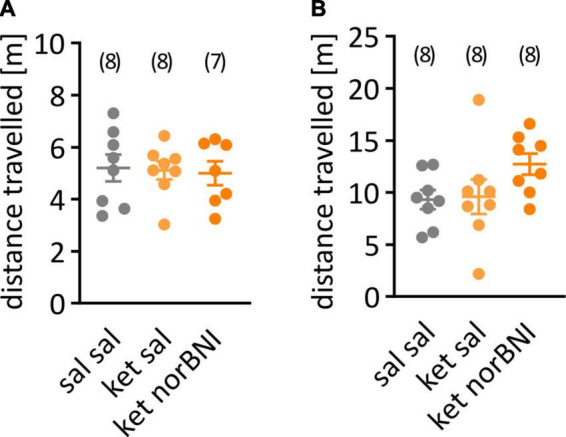
Locomotor activity during the habituation to the partition and open field tests. **(A)** Distance traveled during the habituation to the partition apparatus. **(B)** Activity during habituation to the open field. The group that received only saline is labeled “Sal sal”, ketamine followed by saline “ket sal”, and ketamine followed by norbinaltorphimine “ket norBNI”. Each dot represents an individual mouse. The bar and whiskers correspond to mean and SEM respectively. Group sizes are indicated above the graph.

**FIGURE 3:**
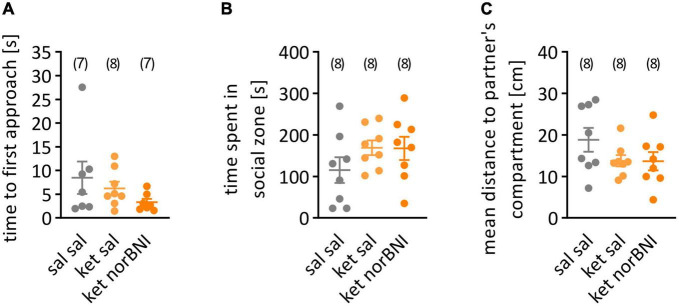
Social interaction in partition test. **(A)** time to first approach, **(B)** time spent in social zone, **(C)** mean distance to partner’s compartment. “Sal sal” corresponds to treatment with saline throughout the procedure, “ket sal” represents subchronic ketamine treatment and then saline injection before the test, and “ket norBNI” is ketamine treatment followed by single injection of norbinaltorphimine. The lines represent the mean and SEM. The numbers above the graphs indicate group sizes.

**FIGURE 4:**
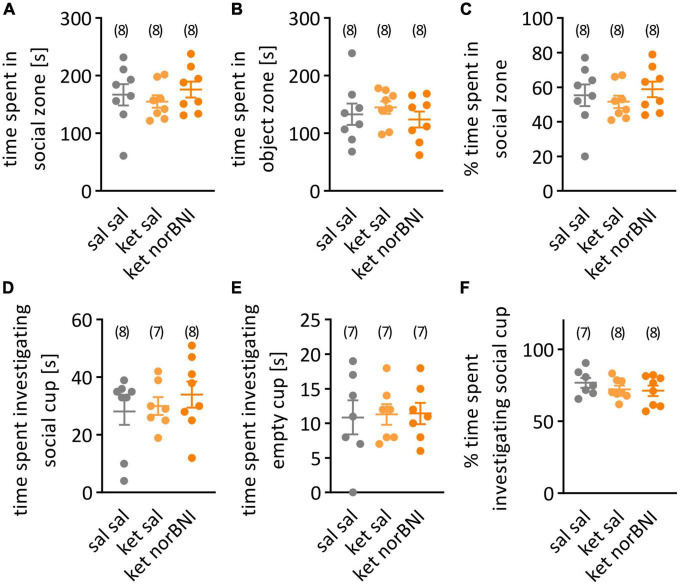
Social interaction in the open field. **(A)** Time spent in in the half of the cage with the partner mouse under the wire cup. **(B)** Time spent in the empty-cup zone. **(C)** Percent time spent in the social zone. **(D)** Time spent investigating the wire cup containing a partner mouse. **(E)** Time spent investigating empty cup. **(F)** Percent time spent investigating social cup. Groups are labeled as follows: “Sal sal” is the control group with only saline injections, “ket sal” corresponds to ketamine treatment and then saline, and “ket norBNI” is ketamine followed by norbinaltorphimine. Results are shown as mean and SEM. The numbers above the graphs indicate group sizes.

Statistical analysis was performed using GraphPad Prism 9. Before group comparisons, the data were scanned for outliers using the Grubbs’ test. When an outlier result was identified, it was removed from the specific comparison, but left in all other comparisons. Outlier measurements were identified in the following variables: distance moved in the partition test (Figure 2A, ket norBNI group), time to first approach in the partition test (Figure 3A, sal sal and ket norBNI groups), time spent investigating social cup in the open field test (Figure 4D, ket sal group), time spent investigating empty cup in the open field test (Figure 4E, all groups), percent of time spent investigating social cup (Figure 4F, sal sal group). Departure from normality was tested using the Shapiro-Wilk test. In cases where significant departure from normality was detected, results were analyzed using nonparametric tests: Kruskal-Wallis followed *post-hoc* with the Dunn test for multiple group comparisons, or alternatively Wilcoxon test for within-subject comparison (social memory). In cases where no departure from normality was detected, data were analyzed with one-way ANOVA followed *post-hoc* with Tukey’s HSD. Alpha level was set to 0.05. One mouse from the “ket norBNI” group escaped the cage during the habituation phase in the partition test, and was excluded from the analysis of locomotor activity during partition test.

## Results

First, we tested whether sub-chronic ketamine administration affected locomotor activity in C57BL/6N mice, as it does in Swiss or C57BL/6J mice. Locomotor activity was studied 5 and 8 days after the last ketamine injection. Ketamine treatment, alone or combined with norbinaltorphimine injection, did not affect distance traveled in the partition test [Figure 2A, *F*_(2,20)_ = 0.046, *p* = 0.955] or in the open field [Figure 2B, *F*_(2,21)_ = 0.41, *p* = 0.669].

In order to determine if sub-chronic ketamine administration causes impairment in social interactions in C57BL/6N mice, similar to the C57BL/6J strain (Sultana and Lee, 2020), the partition test was performed. Three behavioral parameters were assessed: time to first approach to partner’s compartment, time spent in the social zone, and mean distance to partner’s compartments. No statistically significant effects of ketamine or ketamine plus nolbinaltorphimine treatment were observed in the time to first approach (Figure 3A, *H* = 2.75, *p* = 0.262), the time spent in the social zone of the apparatus [Figure 3B, *F*_(2,21)_ = 1.38, *p* = 0.273] or the mean distance from the partner’s compartment [Figure 3C, *F*_(2,21)_ = 1.77, *p* = 0.195].

Finally, in the open field test, time spent in social and object zones as well as time spent sniffing both cups (empty and containing a conspecific) were analyzed. Again, none of the parameters significantly differed between the groups [Figures 4A,B, for both time spent in social (A) and object (B) zones, *F*_(2,21)_ = 0.5, *p* = 0.616, Figure 4C, Percent time spent in social zone, *F*_(2,21)_ = 0.52, *p* = 0.599, Figure 4D, Time spent investigating social cup, *F*_(2,20)_ = 0.52, *p* = 0.6024, Figure 4E, Time spent investigating object cup, *F*_(2,18)_ = 0.25, *p* = 0.976, Figure 4F, Percent time spent investigating social cup, *F*_(2,20)_ = 0.79, *p* = 0.465].

## Discussion

Our results show that withdrawal from sub-chronic ketamine administration in the dose that caused psychotic-like symptoms in C57BL/6J mice (Sultana and Lee, 2020) did not cause such symptoms in C57BL/6N animals. No differences were observed in locomotor activity in a novel environment or social interaction with a same-sex conspecific. The same regimen of ketamine administration was shown to reduce locomotor activity in C57BL/6J mice (Sultana and Lee, 2020). Conversely, similar ketamine administration schedules were repeatedly shown to induce hyperactivity in Swiss mice (Monte et al., 2013; Vasconcelos et al., 2015; Choudhury et al., 2016; da Silva Araújo et al., 2017; Onaolapo et al., 2017) and C57BL/6 mice, with sub-strain not specified (Choudhury et al., 2016).

A possible explanation of the lack of significant behavioral alterations in ketamine-treated animals are sub-strain differences. The C57BL/6N and C57BL/6J sub-strains emerged from the two colonies that were separated in 1951. These sub-strains differ genetically as a result of accumulated spontaneous mutations (Simon et al., 2013), which probably underlie differences in baseline measures in blood pressure, metabolism and behavior (Bryant et al., 2008; Simon et al., 2013; Åhlgren and Voikar, 2019). Several reports indicate that C57BL/6J mice show more interest toward novel mouse in social approach test (Åhlgren and Voikar, 2019), enhanced social interaction (Matsuo et al., 2010; Pinheiro et al., 2016), and express a decreased amount of anxiety-like behavior in the open field (Matsuo et al., 2010; Simon et al., 2013; Åhlgren and Voikar, 2019) in comparison to C57BL/6N mice. Average percentage of time spent in the social zone in the open field test in the C57BL/6N control group (Figure 4C, 55%) is lower than in the 6J strain where it was reported to be approximately 70% (Ogundele and Lee, 2018; Sultana and Lee, 2020). Treatment with ketamine (30 mg/kg) was reported to decrease this to 50% (Ogundele and Lee, 2018) or 35% (Sultana and Lee, 2020). Therefore, we don’t interpret the observed lack of difference in social interaction after sub-chronic ketamine treatment in our research as an indication of a floor effect.

Furthermore, the literature indicates that differences in behavior can potentially exist not only between strains and sub-strains themselves, but also arise from the environmental factors such as laboratory environment or derivation from different vendors (Åhlgren and Voikar, 2019). The results presented here demonstrate, that the minor differences between sub-strains and standardized environments are sufficient to dramatically affect the behavioral phenotype. Importantly, we observed no ketamine-induced impairments, thus the putative differences appear to offer resilience to the psychotomimetic effects.

Additionally, a second factor that may contribute to the lack of appreciable ketamine effects in this study could be the dose of ketamine used or the treatment length. We applied the same ketamine treatment scheme (5 days, 30 mg/kg per day) that was described before to induce wide-range of psychotic-like behavioral alterations in C57BL/6J mice (Ogundele and Lee, 2018; Sultana and Lee, 2020). However, earlier studies on Swiss mice usually used either higher doses of ketamine (Chatterjee et al., 2011; Choudhury et al., 2016) or longer treatment schedules (Featherstone et al., 2012; Monte et al., 2013; Ben-Azu et al., 2018). In one study that included both Swiss and C57BL/6 subjects, authors noted that C57BL/6 showed strong ketamine-induced impairments at doses that had no apparent effects in Swiss mice (Choudhury et al., 2016). In this study Swiss mice were given 100 mg/kg per day and C57BL/6 mice 70 mg/kg (Choudhury et al., 2016). It is possible that administration of 60 mg/kg ketamine for 5 days would induce expected behavioral deficits in C57BL/6N mice too. Alternatively, longer treatment with lower ketamine dose would be necessary. Chatterjee et al. (2011) mention that in Swiss mice the enhancement of immobility in forced swim test was observed after 10, but not after 5, days of ketamine administration (however, the data obtained after 5 days are not shown). We cannot exclude the possibility that social impairment in C57BL/6N mice would emerge after a longer ketamine treatment.

It should be also noted that in this study ketamine treatment was administered in adult C57BL/6N animals, while in the previous report where the same treatment regimen affected social behavior of C57BL/6J mice, animals received ketamine in the late adolescent period (post-natal days 45–60) (Sultana and Lee, 2020). Speculatively, the effects of ketamine treatment could stem from disrupted late brain development, and thus the age of treatment could be critical for the phenotype to develop. While such possibility cannot be entirely excluded, it appears unlikely. In mice, behavior of late adolescent (i.e., sexually mature) males is, in most respects, similar to the behavior of adults (Bell, 2018; Reiber et al., 2022). Moreover, sensitive periods for the development of social behavior, i.e., periods when environmental disturbances influence later behavior most, were described to occur much earlier in the mouse ontogeny (Dyer and Southwick, 1974; Makinodan et al., 2012; Bicks et al., 2020).

Finally, the apparent difference in the response of the C57BL/6N and C57BL/6J sub-strains to ketamine treatment resembles their different sensitivity to addictive substances. The C57BL/6N shows smaller increase in motor activity after acute or repeated treatment with psychostimulants (Kumar et al., 2013), weaker response to nicotine-induced phenotypes (Akinola et al., 2019) and also differences in sensitivity to the effects of alcohol (Crabbe, 1983; Seemiller et al., 2022). The extent of the differences in drug sensitivity is surprising, considering the very small differences in genotype (Simon et al., 2013), nevertheless, it cautions that no assumption of equivalence in drug-induced phenotypes between the two sub-strains should be made.

## Conclusion

In summary, we have shown that sub-chronic administration of high ketamine dose in a scheme that causes psychotic-like behavior in C57BL/6J mice does not induce negative schizophrenia-like symptoms in C57BL/6N mice. We conclude that there are sub-strain differences in the behavioral reaction to sub-chronic ketamine administration in mice.

## Data availability statement

The datasets presented in this study can be found in online repositories. The names of the repository/repositories and accession number(s) can be found below: https://figshare.com/ and https://doi.org/10.6084/m9.figshare.15022764.v1.

## Ethics statement

This animal study was reviewed and approved by the 2nd Local Ethics Committee, Krakow, Poland.

## Author contributions

ZH: conceptualization, methodology, formal analysis, investigation, writing—original draft, writing—review and editing, visualization, project administration, and supervision. KM: investigation and writing—original draft. MK and MC: formal analysis. JRP: conceptualization, methodology, writing—review and editing, project administration, supervision, and funding acquisition. All authors contributed to the article and approved the submitted version.
